# The Psychological Effects of Strength Exercises in People who are Overweight or Obese: A Systematic Review

**DOI:** 10.1007/s40279-017-0748-5

**Published:** 2017-06-01

**Authors:** Gill A. ten Hoor, Gerjo Kok, Gjalt-Jorn Y. Peters, Tim Frissen, Annemie M. W. J. Schols, Guy Plasqui

**Affiliations:** 1 Department of Human Biology, Nutrition and Translational Research in Metabolism, Maastricht University Medical Centre+, Maastricht, The Netherlands; 2 Department of Work and Social Psychology, Maastricht University, P.O. Box 616, 6200 MD Maastricht, The Netherlands; 3 Department of Methodology and Statistics, Open University of the Netherlands, P.O. Box 2960, 6401 DL Heerlen, The Netherlands; 4 Department of Respiratory Medicine, Research School NUTRIM, Maastricht University Medical Centre, P.O. Box 616, 6200 MD Maastricht, The Netherlands; 5 Department Of Human Biology, Maastricht University Medical Center, P.O. Box 616, 6200 MD Maastricht, The Netherlands

## Abstract

**Background:**

Overweightness and obesity represent a high burden on well-being and society. Strength training has positive effects on body composition and metabolic health for people who are overweight or obese. The evidence for psychological effects of strength exercises is unclear.

**Objective:**

The aim of this study was to assess the psychological effects of strength exercises for people who are overweight or obese.

**Methods:**

Relevant literature was identified by use of the PubMed and PsycINFO databases. For each study, effect sizes and corresponding variance estimates were extracted or calculated for the main effects of strength exercises on psychological outcomes.

**Results:**

Seventeen studies were included. There was almost no overlap among the various measures of psychological constructs. The constructs were ordered into eight broad categories. Meta-analytical techniques revealed substantial heterogeneity in effect sizes, and combined with the low number of effect size estimates for each outcome measure, this precluded meta-analysis. Organization of the data showed that the evidence base so far does not show convincing effects of strength training on psychological outcome measures. Some weak effects emerged on self-efficacy, self-esteem, inhibition, and psychological disorders (e.g., anxiety and depression). No additional or comparable effects to other interventions were found for mood, outcome expectations, quality of life, and stress.

**Discussion:**

The main finding of this review is that despite a strong theoretical basis for expecting positive effects of strength training on psychological outcomes, the literature shows a large gap in this area. The existing research does not show a clear picture: some positive results might exist, but there is a strong need to accumulate more evidence before drawing conclusions.

## Key Points

**Table:** 

The literature on the effects of strength exercises on psychological outcomes is fragmented in terms of outcome measures and shows considerable heterogeneity.
Synthesis of the outcomes shows weak effects of strength exercises on psychological outcomes.
This incompleteness of the evidence base, in combination with the strong theoretical basis for assuming positive effects of strength exercises on psychological outcomes, implies an urgent need for more research.

## Introduction

Overweightness and obesity are worldwide problems with high costs to society and personal well-being [1, 2]. Being physically active can both prevent and decrease overweightness and obesity [3]. The substantial public health benefits of successfully promoting exercise in these populations has resulted in a multitude of behavior change interventions targeting exercise. However, meta-analyses showed that few such attempts yielded the desired results [4–7]. It was recently argued that these failures may be partly explained by the wrong choice of behavioral change [8, 9], i.e. many exercise interventions often promote aerobic exercises (see next paragraph). People who are overweight or obese differ from non-overweight people in that they have more weight to carry during exercises. In an absolute sense, this means that, in addition to a higher fat mass, they have higher muscle mass compared to the non-overweight people [10].

These biological differences have not yet been translated to the health psychology field. They could be of substantial benefit to intervention development efforts as health psychology theories make interesting predictions about these dynamics. For example, whereas people who are overweight or obese are unlikely to have mastery experiences when engaging in aerobic exercise, this is much more likely when they engage in strength exercise. Therefore, strength exercise will likely result in increased self-efficacy (e.g., Bandura [11] and Kelder et al. [12]). Self-efficacy is an important determinant of health behavior [13], including exercise behavior [14]. Similarly, when exercising together with non-overweight peers, the superior performance of people who are overweight on strength exercises can foster positive outcome expectations [9].

We previously proposed to combine these biological and psychological insights to argue in favor of exercises for people who are overweight or obese focusing on strength, suggesting that: (1) people who are overweight or obese are stronger (in the absolute sense) and better at (absolute) strength exercises compared with normal weight people; (2) strength exercises are easier for people who are overweight compared with aerobic exercises, and therefore compliance is greater; (3) people who are overweight may enjoy strength exercises, by being better at strength exercises than aerobic exercises than normal-weight people, facilitating long-term behavior change; (4) strength exercises have beneficial effects on the body composition of people who are overweight or obese and thus on metabolic and cardiovascular health [8, 9].

As a first step towards considering strength exercises in health behavior change interventions targeting overweightness and obesity, it is necessary to systematically map what is known about the differential psychological consequences of strength versus aerobic exercise. Indeed, strength training does have positive effects on body composition and health for people who are overweight or obese [15], but the evidence for positive psychological effects is limited (e.g., Lubans et al. [16]) and still unclear at present (for an extensive overview, see Lloyd et al. [17]) [18]. In an earlier review by Schranz and colleagues [19], the effects of strength training on strength, body composition, and psychosocial status were examined in adolescents who are overweight or obese. In their review, four papers that focused on psychological outcomes were included, but in none of these four studies was the independent effect of strength training on psychological outcomes reported (i.e., two studies compared a resistance + aerobic + diet intervention with a diet intervention; one study examined the effects of a combined resistance + aerobic + diet + behavioral therapy intervention vs. a no-intervention control group, and one study examined the time effects of a combined resistance + aerobic + behavioral therapy intervention). Additionally, the limited number of studies and conflicting findings prevented a definitive conclusion. The aim of the current systematic review was to assess the independent psychological effects of strength training or strength exercises for people who are overweight or obese.

## Methods

### Data Sources and Search Strategy, Study Selection, and Data Extraction

For the literature review, no restrictions were made regarding year of publication, language of the manuscript (although all manuscripts found were in English), or design of the study. Because of the expected limited number of studies in this specific area, three criteria were originally used for inclusion. Initially, we aimed to develop a search strategy to locate all studies in (1) people (all ages and both sexes) who are overweight or obese that reported the effect of (2) strength exercises on (3) at least one psychological construct. However, for the literature search, this last criterion turned out to be not feasible, because the psychological outcomes were too varied depending on the underlying theoretical concept. Given our aim of identifying any effects that strength exercises may have on psychological outcomes, it proved impossible to capture this last criterion in query terms without running a considerable risk of excluding potentially relevant literature. Therefore, we used the first two criteria and then selected papers that mentioned any psychological concepts, first based on title and abstract and later on full text (see Table 1). Only studies that reported the independent effect of strength training on psychological outcomes in overweight or obese people were included. No other restrictions were applied.

**Table 1:** Search terms used in the systematic review and meta-analysis

((overweig*) OR (obese) OR (obes*) OR (obesity) OR (overweight) OR (weight status) OR (adipos*))
AND
((strength*) OR (Strength) OR (resistance) OR (resist*) OR (weight-lifting) OR (weight lifting) OR (weight bearing) OR (weight-bearing)) AND ((program*) OR (intervention) OR (train*) OR (exercis*))

**Fig. 1:**
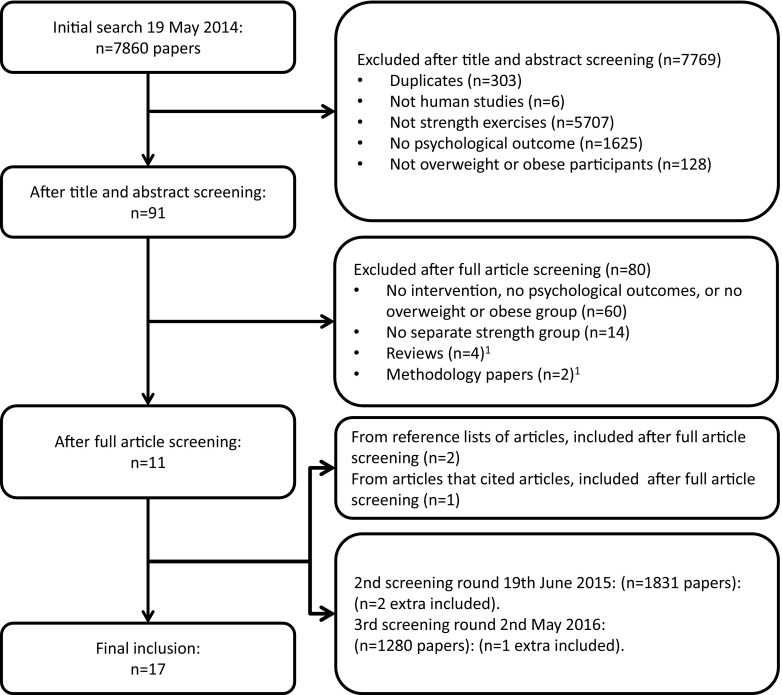
Flowchart of the literature search progress

Relevant literature was identified using the PubMed and PsycINFO databases (first data search on 19 May 2014). In the first screening round (*N* = 7860) two screeners (GH and GK) identified 14 papers that met the eligibility criteria (see Fig. 1 for a flowchart showing the literature search progress). In a second and third screening round a total of three additional studies were found. The final number of included studies was 17. In the Supplementary Material at https://osf.io/8jbaz/ (Open Science Framework), a detailed list of all initial paper titles and abstracts can be found, including why papers were systematically excluded, together with the PRISMA checklist [20].

### Study Quality and Categories

To acknowledge study quality and simultaneously take into account the intervention administered in the control group [21], we divided studies into five categories (see also Table 2). Studies in category I (a no-intervention control group compared to strength training) can answer the question of whether strength training has an effect on psychological outcomes. Studies in category II (an active control group vs. the same active control group plus strength training) can answer the question of whether strength training has added value over and above the active control group intervention. Studies in category III (an active control group vs. strength training) can answer the question of how strength training performs compared to the active control group intervention (e.g., diet or aerobic training). Category IV (an active control group (e.g., aerobic plus diet) versus strength training plus another active component (e.g., diet) can answer the question of how strength training performs compared to a given active component, when both are combined with another active component. Category V (studies lacking a control group, i.e., pretest–post-test designs) can provide very weak evidence for an effect of strength training over time, and was mainly included for the sake of completeness. To assess study quality, an additional risk of bias assessment was performed using the Effective Public Health Practice Project Quality Assessment Tool [22]; see also the Supplementary Material).

**Table 2:** Study type categorization

Category	Strength training group		Comparison group	Example		
I	Strength	vs.	Passive control	Strength training	vs.	No-intervention control
II	Strength + active control	vs.	Active control	Strength training + diet	vs.	Diet
III	Strength	vs.	Active control	Strength training	vs.	Diet
IV	Strength + active control I	vs.	Active control I + active control II	Strength training + diet	vs.	Diet + aerobic training
V	Strength	vs.	No control	Strength training	vs.	–

### Measures of Psychological Outcomes

There was great variation in the psychological terminology used in the included studies. To establish which constructs could be aggregated, GtH extracted the variables and their operationalization from the included papers.

To determine which psychological outcome measures could be aggregated, two authors (GK and GJP) indicated which construct they thought was being measured. To establish this, they consulted the papers’ methodology sections where necessary. After this coding phase, two discussion rounds were conducted. In the first, both coders, facilitated by a third (GtH), discussed the terminology used where there were minor deviations [e.g., one author used “mood (inverted)” and the other “negative mood,” but the same variables were coded (90% consensus)]. In the second discussion round, more fundamental differences were discussed and resolved. After consensus was achieved, the psychological outcomes were ordered into eight broad categories: disorders (e.g., anxiety and depression), inhibition, mood, outcome expectations, quality of life, self-efficacy, self-esteem, and stress (see the Supplementary Material for the coding sheets). The resulting spreadsheet was then imported into R [23] for further analysis using metafor [24].

### Analyses

For each study, effect sizes as well as corresponding variance estimates were extracted or calculated for the main effects of strength exercises on strength (as strength interventions are often focused on improvements in strength) and on psychological outcomes. Most studies used split-plot designs where within-subjects pre- and post-tests were combined with a between-subjects manipulation (see also Table 3). In such cases, computation of the effect sizes’ variance estimates requires the correlation between pre- and post-test measures [25], which was not reported by any of the papers. We therefore computed three types of variance estimates, assuming correlations of 0.3, 0.5, and 0.7 (corresponding to the qualitative labels for effect size as tentatively suggested by Cohen [26]). All analyses were therefore conducted three times. The results for the correlation estimate of 0.3 are reported, supplementing these reports with discussion of diverging outcomes where these occur.

**Table 3:** Study characteristics

Study	Study design		BMI, mean (SD)	Outcome (questionnaire)	*n* (F) age, years {range or [mean (SD)]}	Study duration	Category^a^
	Strength component	Comparison					
Davis [28]	Standard behavioral weight loss program + strength training	(1) Standard behavioral weight loss program (2) Standard behavioral weight loss program + mindfulness	All: 32.9 (3.7)	Eating behavior, self efficacy for physical activity and weight loss, exercise beliefs, body image, mindfulness	71 (63) [25–39.9]	24 weeks	II
Fonzi [33]	Standard behavioral weight loss program + home based strength training	Standard behavioral weight loss program	All: 33.3 (3.5)	Health related quality of life	48 (42) [18–55]	12 weeks	II
Ghroubi et al. [34]	Treadmill training + dietary advice + strength training	(1) No intervention control (2) Treadmill training + dietary advice	All: 37.2 (5.2)	Psychological impact of obesity, quality of life	83 (70) [18–60]	8 weeks	II
Goldfield et al. [29]	(1) Strength (2) Strength + aerobic	(1) Aerobic training (2) No intervention control	All: 34.6 (4.5)	Body image, physical self–perceptions and global self–esteem, mood	304 (213) [14–18]	24 weeks	I, II, III
Lau et al. [35]	Dietary education and modification + strength training	Dietary education and modification	Intervention: 30.4 (4.7) Control: 29.0 (5.1)	Depression and anxiety	37 (25) [10–17]	6 weeks	II
Levinger et al. [31]	(1) HiMF + strength training (2) LoMF + strength training	(1) HiMF no intervention control (2) LoMF no intervention control	Intervention: (1) 31.6 (4.4) (2) 23.8 (3.1) Control: (1) 30.0 (3.7) (2) 24.3 (3.4)	Self perceived physical and mental quality of life	55 (27) [40–69]	10 weeks	I
Levinger et al. [36]	Acute session of strength training in (1) male non obese (2) male obese (3) female non obese (4) female obese	–	Group (1) 24.2 (0.9) (2) 31.0 (0.9) (3) 21.6 (0.8) (4) 30.6 (1.2)	Positive well-being, psychological distress and fatigue, health related quality of life	45 (23) [40–69]	Acute session of strength training	V
Levinger et al. [37]	(1) HiMF + strength training (2) LoMF + strength training	(1) HiMF no intervention control (2) LoMF no intervention control	All: 27.7 (0.7)	Depressed mood, physical health, mental health	55 (27) [40–69]	10 weeks	I
Martins et al. [38]	Strength training	(1) No intervention control (2) Aerobic training	Intervention: 30.1 (4.6) Control (1) 29.0 (4.4) (2) 29.8 (4.4)	Mood states—depression, tension-anxiety, fatigue, vigor-activity, anger-hostility, confusion-bewilderment	78 (48) [65–95]	16 weeks	I, III
Messier et al. [27]	Caloric restriction group + strength training	Caloric restriction group	Intervention: 32.6 (4.9) Control: 32.2 (4.6)	Body esteem, self-esteem, stress, dietary restraint, disinhibition, hunger, quality of life, self-efficacy, perceived benefits, perceived risks	137 (137) [58 (5)]	25 weeks	II
Plotnikoff et al. [39]	Strength training	No intervention control	Intervention: 25.6 (7.8) Control: 38.5 (8.1)	Social cognitions	48 (32) [55 (12)]	16 weeks	I
Sarsan et al. [40]	Strength training	(1) No intervention control (2) Aerobic training	Intervention: 33.73 (2.92) Control: (1) 35.54 (4.98) (2) 35.38 (4.98)	Ratings of mood	60 (60) [20–60]	12 weeks	I, III
Schranz, et al. [18]	Strength training	No intervention control	Intervention: 32.2 (4.3) Control: 32.6 (5.0)	Self- efficacy, physical self-worth, self-esteem	56 (0) [13–17]	24 weeks	I
Wadden et al. [32]	(1) Diet + strength training (2) Diet + aerobic + strength training	(1) Diet (2) Diet + aerobic training	All: 36.5 (5.1)	Appetite, mood	128 (128) [41.1 (8.6)]	48 weeks	II, IV
Wicker et al. [30]	Strength training	–	25.9 (4.74)	Satisfaction	10,386 (7,260) [46.4 (15.4)]	4 weeks	V
Williams et al. [41]	Strength training	–	33.1 (3.8)	Outcome expectancy, behavioral expectation, self-regulation, resistance training strategies, perceived satisfaction, intention	123 (91) [not stated]	24 weeks	V
Yu et al. [42]	Diet + strength training	Diet	Intervention: 25.6 (3.2) 24.7 (3.0)	Physical self-concept	82 (28) [8–11]	6 weeks	II

*BMI* body mass index, *SD* standard deviation, *F* female, *HiMF* high metabolic risk factor, *LoMF* low metabolic risk factor

^a^For category labels, see Table 2

Where studies reported multiple effect size estimates for variables that were coded as the same variable [e.g., “disinhibition” and “hunger” in Messier et al. [27] were both coded as “inhibition (inverted)”], these were first aggregated to obtain one estimate per variable per study. For these intra-study meta-analyses as well as the final between-study meta-analyses, random effects meta-analyses were conducted using the metafor package’s restricted maximum-likelihood estimator [24]. Heterogeneity was estimated using *τ* ^2^ (estimated between-study variance), *I* ^2^ (the proportion of variability in effect sizes due to heterogeneity rather than error), *H* ^2^ (total variability compared to sampling variability) and *Q* (the *χ* ^2^ test for heterogeneity), and forest and funnel plots were generated for each meta-analysis and are available in the Supplementary Material.

We identified “positive effects” of strength exercises in people who are overweight or obese as occurring when psychological constructs changed in the desired direction (e.g., increase in self-efficacy or decrease in psychological distress).

## Results

### Study Selection and General Characteristics

In total, 17 studies were included in the systematic review (Fig. 1). Based on our risk of bias assessment, the study quality of 13 papers was rated as “moderate” and four papers were rated as “weak” (see the Supplementary Material). Study characteristics are listed in Table 3. The number of participants in the different comparisons ranged from 32 [28] to 304 [29], with one extreme of 10,386 participants [30]. The intervention period ranged from an acute session of strength exercises [31] to 48 weeks’ training [32]. Seven studies included a comparison between strength training and a no-intervention control group (category I; see also Table 2 for examples). Eight studies included comparisons between an active control group (e.g., diet) and the same control group plus strength training (category II). Three studies compared strength training to aerobic training (i.e., an active control group—category III). One study compared strength training plus diet to aerobic training plus diet (category IV). Finally, three studies employed a pretest–post-test design (category V). Thirteen studies were in adults. All studies included a specific group of people who were overweight or obese (see Table 3).

### Study Outcomes: Psychological Benefits

The 17 included studies had many different psychological outcomes. These are summarized in Table 4.

**Table 4:** Psychological outcomes per study

Study	Intervention (resistance training group)	Comparison group	Psychological outcomes
Davis [28]	R + Ae + D	Ae + D Ae + D + mind-fulness	Eating behavior scores improved for all groups, without differences in groups. Intention-to-treat analyses show that the mindfulness group had greater scores compared to the standard behavioral weight loss program group Mindfulness improved over time, but did not significantly differ between groups Self-efficacy for physical activity when tired, when on vacation, and eating self-efficacy improved for all groups, but did not significantly differ between groups Dietary restraint increased for all groups, without significant differences between groups Body image improved over time for appearance evaluation, fitness orientation, health evaluation, health orientation, illness orientation, body-areas satisfaction, self-classified weight over time, and overweight preoccupation Differences between groups were found only for health evaluation In all groups a significant decrease in expected barriers for physical activity was found without differences between groups. A significant group × time interaction was found for the time barrier Outcome expectations increased most in mindfulness and resistance training group The mindfulness group had much higher expectations that body image will improve with exercise compared to the SBWL group
Fonzi [33]	R + Ae + D	Ae + D	No significant differences were found over time for social functioning, bodily pain, mental health, “role emotional” Significant increases were found for “role physical,” vitality, and general health (trend for physical functioning). No differences between groups were found
Ghroubi et al. [34]	R + Ae + D	Ae + D No intervention	All stress test parameters improved in intervention groups but not in control group Psychological status (anxiety, depression, and quality of life) improved in intervention groups but not in control group
Goldfield et al. [29]	R R + Ae	No intervention Ae	Time, but no group × time, effects on body image Time, but no group × time, effects on anger and depression Significant effects on vigor (group × time) No effects on confusion, fatigue or tension Time, but no group x time, effects on self-perceived skills, and perceptions of physical self-worth Perceived physical condition, global self-esteem and strength were improved for the R&AE group vs. control group
Lau et al. [35]	R + D	D	Non-significant improvement was found in anxiety or depression in both groups No difference was found between the two groups for anxiety or depression
Levinger et al. [31]	R	No intervention	Training did not improve psychological outcomes in the LoMF group Training increased perception of both physical and mental health in the HiMF group compared to the control group Training improved scores on physical functioning, general health, social functioning in the HiMF training group Self perceived bodily pain got worse in the LoMF training group and improved for the HiMF training group Self-perceived physical health improved more in the HiMF training group compared to the LoMF training group
Levinger et al. [36]	R	–	In women, exercise increased positive well-being after exercise Positive well-being in obese women tended to improve (*p* = 0.059) Exercise did not change perception of psychological distress of fatigue in women (within and between) Fatigue increased after exercises more in non-obese men compared to obese men No changes in positive well-being of psychological distress were found in men
Levinger et al. [37]	R	No intervention	At baseline, no differences in depression scores between LoMF groups. The HiMF training group had a higher depression score at baseline compared to the HiMF control group After training, depression score was improved in the HiMF training group compared to the HiMF control group (no such results were found in the LoMF groups)
Martins et al. [38]	R	Ae No intervention	Mood states changed over 16 weeks in the control group (more confusion) and strength training group (positive change in vigor) Furthermore, no differences were found after 15 weeks in depression, tension, fatigue, and anger
Messier et al. [27]	R + D	D	Both groups improved for total body esteem, body esteem subscales, dietary restraint, disinhibition, hunger, quality of life subscale for health perceptions, and self-efficacy No additional effects of resistance training on psychological factors were found
Plotnikoff et al. [39]	R	No intervention	After 16 weeks resistance intention items significantly increased in the resistance training group compared to the control group After 16 weeks scheduling self-efficacy was higher in the intervention group vs. control Task and barrier self-efficacy, and health-related quality-of-life scores did not change significantly between groups For individuals who completed at least 2/3 of the intervention, significant gains in task, schedule, and barrier self-efficacy were found compared to individuals who completed less than 2/3 of the intervention
Sarsan et al. [40]	R	Ae No intervention	Both exercise groups improved in depression score. Only the aerobic exercise group changed significantly compared to the control group
Schranz, et al. [18]	R	No intervention	Significant differences were found between intervention and control group at 3 and 6 months in exercise self-efficacy No significant differences between groups for resistance training beliefs (but large difference for the subscale confidence) Trends were found for physical self-worth (not statistically significant between groups) At 3 and 6 months, intervention group had higher global self-esteem compared to control group
Wadden et al. [32]	R R + Ae + D	D Ae + D	No significant differences among conditions at any time were found in changes in hunger, satiety, preoccupation with food, or intensity of food cravings Mood changed over time in all groups. No significant differences among conditions were found in BDI scores No significant differences among conditions in changes on any of the profile of mood states. In all conditions increases were found in vigor, and decreases in fatigue
Wicker et al. [30]	R	–	Increases in life satisfaction Increases in health satisfaction
Williams et al. [41]	R	–	Resistance training intervention had significant effects on change in behavioral expectation, self-regulation, and perceived satisfaction but not outcome expectancies
Yu et al. [42]	R + D	D	Confidence in strength increased significantly in both groups after intervention The diet-and-strength training group increased significantly in self-concept of endurance compared to the diet-only group

*Ae* aerobic exercise intervention, *BDI* Beck Depression Inventory, *BMI* body mass index, *D* diet intervention, *HiMF* high metabolic risk factor, *LoMF* low metabolic risk factor, *R* strength or resistance exercise intervention, *SBWL* standard behavioral weight loss program

Based on the available data, for two studies [32, 34] no effect sizes could be calculated, and, therefore, these were not included in the meta-analysis. One additional study [36] was excluded for meta-analysis, as this study examined the acute effects of one strength exercise session. For all other studies effect sizes were calculated based on pre- and post-test means, standard deviations (SDs) and *n* values in both the strength-exercise group and the comparison group. In one study [35] effect sizes were available. Study outcomes were divided into the five major study types and eight major outcome categories. All individual effect sizes and forest and funnel plots can be found in the Supplemental Material. Note that although the literature contained reports of the effect of strength training on eight different psychological variables, few studies were available for each variable; and as the various studies provided data to answer different research questions, few studies were available for meta-analysis. This small number of studies for meta-analysis made heterogeneity hard to assess. Effect sizes seemed quite consistently heterogeneous for the exercises’ effects on strength (see Supplementary Material). Heterogeneity varied from 0–100%, with *p* values from <0.001 to 1 (see also the Supplemental Material).

The current state of the literature means that it is unclear how results from the meta-analyses should be interpreted. Therefore, the outcomes will be discussed qualitatively. We have, however, used the meta-analysis to generate diamond plots to aid interpretation of the current evidence base.

The diamond plots show that all effects are weak, but most of them are in a positive direction (i.e., strength training has a possible positive influence on psychological outcomes). Some weak effects emerged on self-efficacy, self-esteem, and psychological disorders (e.g., anxiety and depression), but only compared to a no-intervention control group [first diamond plot (category I)]. The second diamond plot (category II) shows that strength exercises have possible favourable additional effects on psychological disorders, self-esteem, and inhibition when combined with another active component, but that they are weak and have no additional effects on stress, self-efficacy, quality of life, or outcome expectations. In the third diamond plot, strength exercises were compared with other interventions [e.g., diet or aerobic exercises (category III)], showing that strength has possible positive effects on self-esteem but no stronger effects than diet or aerobic interventions on psychological disorders, quality of life, or mood. For the fourth study type [an active control group (e.g., aerobic plus diet) vs. strength training plus another active component (i.e., diet)], no data were available [32]. For the fifth study type (pre–post-test design without a control group), positive time-effects for strength training were found for perceived well-being [31], health and life satisfaction [30], and behavioral expectation, self-regulation, and perceived satisfaction [35]. The study examining the acute effects of strength exercises showed some positive effects on well-being, but the results were inconclusive [36]. Subclassification by age (i.e., under 18 years and over 18 years) showed no clear differences in results (see the Supplemental Material).

## Discussion

**Fig. 2:**
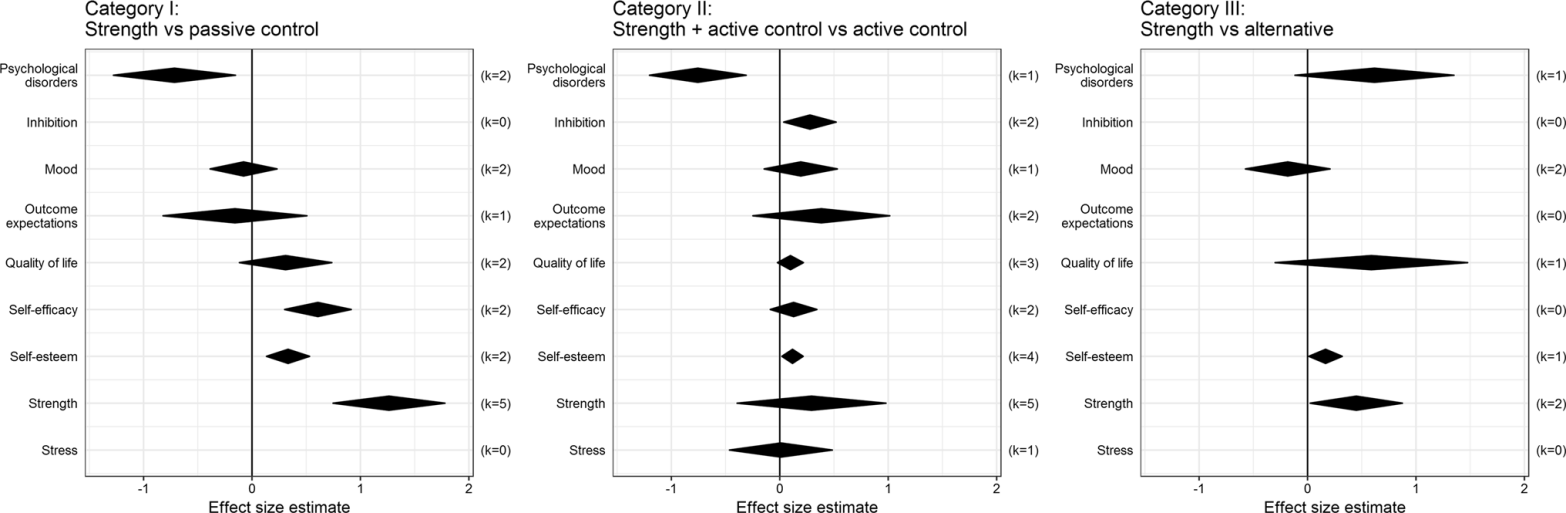
Effects of strength exercises on psychological outcomes: pooled effect sizes obtained from meta-analyses

Seventeen studies were included in this systematic review investigating the psychological effects of strength training in people who are overweight or obese. Strength training for people who are overweight or obese had small positive effects on various psychological outcomes when compared to a no-intervention control group, but these effects were often comparable to those of aerobic and diet interventions (Fig. 2).

The various studies included in this review reflect a combination of high heterogeneity and a low number of existing studies. This reflects the unfortunate state of the literature, and is the main reason why our conclusions, despite our use of meta-analysis to aid interpretation, are tentative.

The two common responses to this combination of heterogeneity and low number of studies are (1) to conduct separate analyses to eliminate heterogeneity per analysis and (2) to combine outcome measures or study methodologies to maintain the number of studies in each analysis. It is difficult to conduct these responses at the same time and they are not reconcilable with each other. We therefore decided to report our analyses as they are. There is no evidence or theory to guide us to an “objectively optimal” solution, and given the current state of the literature, it will take some time before such guidance becomes available. The other consideration is that conducting multiple analyses sharply increases the probability of encountering statistical artifacts (e.g., making type 1 errors). We used a meta-analysis to generate diamond plots to aid interpretation of the current evidence base. In addition, we have provided the dataset (i.e., the extracted data), analyses, and output. This will enable other researchers to separate/pool analyses as they see fit given their specific research interests.

Possible hypotheses for similar effects of strength exercises compared to other interventions on psychological constructs are (1) that the proportion of female participants in some studies was quite high, which might have impacted the results, (2) that for people who are overweight their main goal of participating in physical activity, dietary, or combined weight-loss interventions is generally to lose weight [43], and (3) that the strength exercise component in some studies was limited: for example, in the study by Davis et al. [28], participants were provided with strength exercise equipment and laminated exercise cards with descriptions of the strength training exercise that needed to be executed at home.

In strength-training interventions, it is expected that people gain in muscular mass (lean mass), and therefore may not lose much weight despite a reduction in adipose tissue. Most studies in this systematic review reported that body strength improved after strength training compared to a no-intervention or other-intervention group, while body weight or body composition often did not differ significantly between a strength intervention group and comparison group(s). A first possibility for future studies might be to investigate the influence of giving feedback on body composition during strength interventions. Gaining strength, and ultimately obtaining a healthier body composition, might lead to a higher resting metabolic rate, increased total energy expenditure, and a decreased chronic diseases risk [44]. Thus, when participants in a strength-training program become stronger, this should also lead to (long-term) positive changes in body composition and health. However, these positive effects are often not reflected in reported short-term psychological outcomes of strength training as compared to other interventions.

Given that strength exercises performed similarly to alternative interventions, we might conclude that strength exercises are a viable alternative or addition to diet and/or aerobic interventions, but more research is necessary. Pescud and colleagues [43] reported that feedback on body composition is useful as a “surrogate” for feedback on weight loss, which motivated participants to continue participating in strength-training exercises.

While body composition was reported in 10 out of 18 studies, none of these studies indicated that changes in body composition were given as feedback to the participants. As noted in the previous paragraph, giving feedback on body composition could be a form of positive reinforcement to engage in strength exercises. Also, the reported psychological outcomes were mostly clinical outcomes or markers of quality of life. None of the outcomes focused on self-determination, although self-determination concepts are very popular in motivation and intervention studies of exercise behavior [45]. As we noted in Sect. 1, people who are overweight or obese may discover in a strength exercise program that they are stronger than normal-weight people, which may result in their motivation for exercising to become relatively more intrinsic [8, 9]. Measuring self-determination concepts as psychological constructs might give additional information about the effects of exercise training to be considered alongside that obtained from current clinical and quality-of-life measures.

The strengths of this systematic review are the focus on the independent psychological effects of strength training for people who are overweight, the use of meta-analysis, and the contribution to the available evidence for positive self-reported psychological effects of strength training. The weaknesses of this study relate to the limited range of psychological outcomes and the great variation in psychological terminology used in the included studies.

## Conclusions

This review affords three conclusions. The first is that, indeed, strength exercises have possible positive effects on a number of psychological outcome measures in populations of people who are overweight or obese. The second is that these effects seem comparable to and sometimes stronger than those of aerobic and diet interventions. The third and main conclusion is that due to a lack of data both conclusions are provisional. There is a need for more research, and given the positive effects that can be expected based on theory and the promising patterns that seem present in the presently synthesized empirical evidence, the need is urgent. Future studies should include the effect of giving feedback on improved strength and body composition as motivators for strength-training continuation, as well as measure additional psychological outcomes such as self-determination concepts.
